# A Survey on Additional Health Risk Factors for Heroin Users Presenting in Emergency Department (ED) of Chesterfield, North Derbyshire

**DOI:** 10.1192/bjo.2024.515

**Published:** 2024-08-01

**Authors:** Adeel Rauf, Deepak Sirur, Martin Smith

**Affiliations:** Derbyshire Healthcare NHS Foundation Trust, Chesterfield, United Kingdom

## Abstract

**Aims:**

1. To identify various physical and social health characteristics of heroin users to reduce further risks presenting to ED in Chesterfield, North Derbyshire.
To consider whether any characteristics identified could develop a targeted screening tool for enhanced interventions.

**Methods:**

A retrospective review of ED notes was conducted from Chesterfield Royal Hospital using electronic patient records of heroin users who are under the care of Drug and Recovery Partnership (DRP) in Chesterfield, North Derbyshire. We developed a proforma for data collection analysis using Microsoft Excel.

100 patients were chosen over a time interval of one year in which they have had at least one ED presentation.

We looked into Body mass index (BMI), physical health diagnoses, number of presentations to the ED in one year, psychotropic medications, dose of opioid substitution therapy and living circumstances of the attendees. These characteristics were identified in a previous study of local mortality data.

**Results:**

46% of the attendees only presented once in the study interval.

83% of the attendees presented to ED due to a medical reason.

41% of the attendees had raised BMI.

73% of the attendees who attended were on Opioid Substitution therapy (OST). 51% of the attendees were using a dose between 70–100 ml of methadone.

27% of the attendees had co-morbid COPD and Asthma.

47% of the attendees were on prescribed psychotropic agents. 56% of them were prescribed mirtazapine.

44% of the attendees lived alone, 33% with a partner.

**Conclusion:**

1. Based on the sample, 83% of the heroin users presenting to ED in this period of study attended due to physical health concerns.
2. As half of the sample were not serial attenders (46%), it is important that opportunities of assessment for this high-risk group of people are not missed.
3. Nearly three quarters (73%) of the attendees were on prescribed OST, half of those were within optimised dose. This suggests for tighter links between liaison to local drug services to alert presentations with specific consideration of harm reduction interventions, dose optimisation or re-titration onto OST.
4. The data collected over this period supports the development of a pilot screening tool to prioritise enhanced care interventions with a specific focus on harm reduction for a specific group of high-risk heroin users.

